# Satisfaction with Healthcare Received at an Interprofessional Student-run Free Clinic: Invested in Training the Next Generation of Healthcare Professionals

**DOI:** 10.7759/cureus.2282

**Published:** 2018-03-07

**Authors:** Karen B Lu, Bryan Thiel, Christopher A Atkins, Anand Desai, Ariel Botwin, Michael R Povlow, Judith Simms-Cendan, Magdalena Pasarica

**Affiliations:** 1 Medical Student, University of Central Florida College of Medicine; 2 University of Central Florida College of Medicine; 3 Medical Education, University of Central Florida College of Medicine

**Keywords:** interprofessional collaboration, patient survey, quality improvement, student-run free clinic, community health clinics, medical student, medical education, patient-centered outcomes

## Abstract

Most medical schools in the United States have an associated student-run free clinic (SRFC) providing medical care to the underserved population around the campus. SRFCs provide students with opportunities to practice history-taking and diagnosis skills. There have been a few studies that have evaluated patient satisfaction within SRFCs; however, these studies report limited aspects of care within these clinics. This study hopes to determine the levels of satisfaction with clinical staff and operations and to ensure that the medical needs of patients are being met. Results showed that 91% of the patients were satisfied or very satisfied with their overall clinic experience. The highest scoring parameters were “courtesy/respect of staff”, “availability of free or affordable medications”, and “doctor’s knowledge”. Overall, the patients are satisfied with the staff, care, and availability of medicine provided by the Keeping Neighbors in Good Health Through Service (KNIGHTS) clinic. Most patients enjoy participating in the training and education of future physicians and would recommend this clinic to a friend or family member. The lowest satisfaction rates were associated with length of visit and wait time. In the future, SRFCs should work together to assess patient satisfaction in the clinics, identify problem areas, and develop generalizable interventions for improvement.

## Introduction

In 2014, there were over 100 United States (US) medical school-affiliated student-run free clinics (SRFC) serving the uninsured population [1]. This number has nearly doubled since the last survey in 2005 [2]. SRFCs not only provide health care to the underserved populations but also provide patient care experiences for students in training. As with regular free clinics, most SRFCs use an interprofessional approach to patient care. Even though the number of interprofessional SRFCs continues to grow, patient satisfaction amongst SRFCs have been reported only in a few studies. These studies showed high patient satisfaction with care, services provided, staff and provider attitudes, and facility conditions, but low satisfaction with wait time, lack of specialty services, and hours of operation [1, 3-6]. However, these surveys were not validated and did not study all components of the clinic. A study of the comprehensive interprofessional care provided in SRFCs is essential for continuous quality improvement, especially in the setting of high student provider turnovers.

This study evaluates the level of patient satisfaction within multiple aspects of patient care, not previously addressed in the literature, including specialty care availability, students’ and attendings’ knowledge, appointment length, health education, opportunity to assist in the education of future providers, and overall clinic experience, utilizing a internally developed survey. The survey was validated by faculty, staff, students, and clinic patients. The goal of this study was to determine the patient experience at the The Keeping Neighbors in Good Health Through Service (KNIGHTS) Clinic funded by the Diebel Legacy Fund at the Central Florida Foundation, address concerns, and highlight areas of high satisfaction, to continue improving the quality of medical care at this SRFC. This study will also provide valuable data for other SFRCs and free clinics looking to utilize a validated survey to measure patient care.

At the University of Central Florida (UCF) College of Medicine, an interprofessional SRFC, The Keeping Neighbors in Good Health Through Service (KNIGHTS) Clinic funded by the Diebel Legacy Fund at the Central Florida Foundation, was established in 2013. The KNIGHTS Clinic’s goal was to provide patient-centered medical care to the underserved population of Orlando. KNIGHTS Clinic operates at Grace Medical Home (GMH), a free clinic providing care to the uninsured population living 200% below federal poverty level. GMH provided this SRFC with patients off their waiting list, the facility, and other laboratory and medication resources. The KNIGHTS Clinic is staffed by UCF medical and social work students, along with University of Florida pharmacy students, under the supervision of licensed providers affiliated with their respective universities.

## Materials and methods

The study took place between August 2015 and April 2016 at the KNIGHTS Clinic funded by the Diebel Legacy Fund at the Central Florida Foundation. This study was approved by the Institutional Review Board at UCF. All study participants were English-speaking, 18 years of age or older, and patients who received medical care at the KNIGHTS Clinic. The survey (see Appendix) was adapted from published patient satisfaction surveys of SRFCs. The survey consisted of 28 items: 26 multiple choice (of which 15 used a Likert scale) and two free text responses. The survey was content validated using feedback from multiple patients, providers, and student providers. The internal reliability of the survey was calculated using Cronbach’s coefficient alpha with the Statistical Package for the Social Sciences (SPSS) Version 24.0 (IBM Corp., Armonk, NY). The coefficient was found to be 0.77, showing acceptable reliability. Participating patients completed the survey following their appointment.

Descriptive statistics were analyzed for each survey question. A five-point Likert scale was utilized with the following scores: 1 – very unsatisfied, 2 – unsatisfied, 3 – neutral, 4 – satisfied, and 5 – very satisfied. Unanswered questions or those marked "Not Applicable" by individual participants were not included in calculating the mean score.

## Results

Forty-four patients completed the patient satisfaction survey between August 2015 and April 2016. The patients' age ranged from 18 to 65 years, 73% were female, 32% were first time patients, 23% were patients of six months to one year, and 25% were patients of one to two years.

The survey results are presented in Table 1. All survey items scored, on average, above 4, suggesting an overall high satisfaction with clinic services.

**Table 1:** Keeping Neighbors in Good Health Through Service (KNIGHTS) Clinic patient satisfaction survey items and percentages Surveyed KNIGHTS Clinic service parameters expressed as percentages of total responses.

	1 (Very Unsatisfied)	2 (Unsatisfied)	3 (Neutral)	4 (Satisfied)	5 (Very Satisfied)	No response or N/A	Mean rating (SD)
Availability of free or affordable medications	3 (7%)	0 (0%)	1 (2%)	4 (9%)	31 (70%)	5 (11%)	4.64 (1.14)
Availability of primary care and specialists	4 (9%)	0 (0%)	0 (0%)	8 (18%)	29 (66%)	3 (7%)	4.52 (1.23)
Education on disease treatment and prevention	4 (9%)	0 (0%)	2 (5%)	4 (9%)	32 (73%)	2 (5%)	4.5 (1.25)
Education about your medications	4 (9%)	0 (0%)	2 (5%)	5 (11%)	32 (73%)	1 (2%)	4.45 (1.23)
Wait time during this appointment	3 (7%)	0 (0%)	5 (11%)	10 (23%)	26 (59%)	0 (0%)	4.27 (1.13)
Length of time in clinic from arrival to departure	4 (9%)	1 (2%)	3 (7%)	11 (25%)	25 (57%)	0 (0%)	4.18 (1.24)
Overall medical care	4 (9%)	0 (0%)	0 (0%)	4 (9%)	36 (82%)	0 (0%)	4.55 (1.17)
Students’ knowledge	4 (9%)	0 (0%)	1 (2%)	5 (11%)	34 (77%)	0 (0%)	4.48 (1.19)
Doctors’ knowledge	4 (9%)	0 (0%)	0 (0%)	2 (5%)	38 (86%)	0 (0%)	4.59 (1.17)
Treatment by teaching faculty	4 (9%)	0 (0%)	2 (5%)	6 (14%)	32 (73%)	0 (0%)	4.41 (1.21)
Availability of after-hours appointments	4 (9%)	0 (0%)	1 (2%)	5 (11%)	34 (77%)	0 (0%)	4.48 (1.19)
Courtesy/Respect of Staff	4 (9%)	0 (0%)	0 (0%)	4 (9%)	34 (77%)	2 (5%)	4.56 (1.2)
Team approach to care	4 (9%)	0 (0%)	0 (0%)	5 (11%)	35 (80%)	0 (0%)	4.52 (1.17)
Overall clinic experience	4 (9%)	0 (0%)	0 (0%)	6 (14%)	34 (77%)	0 (0%)	4.5 (1.17)

“Students’ knowledge” (4.48 ± 1.19), “courtesy/respect of staff” (4.56 ± 1.2), and “availability of free or affordable medications” (4.64 ± 1.14) were rated, on average, very favorably, with 77% reported being “very satisfied” with these services.

Forty out of 44 respondents (91%) were “satisfied” or “very satisfied” with the “overall clinic experience” and “overall medical care” (Table 1). The lowest satisfaction ratings, earning scores of 4.27 and 4.18, were “length of wait time” and “visit time”, respectively. The overall highest satisfaction score was received by the “availability of free medications”.

Seventy-five percent of patients were satisfied with their improvement in health since joining the clinic. We found that 95% of respondents said that the opportunity to improve the education of future physicians was very important to them, while 2% said that it was somewhat important to them. All respondents indicated that they would recommend the KNIGHTS Clinic to a friend or family member. As for the length of clinic visits, 20% of respondents reported a total visit time lasting over two hours, 30% of patients reported a total visit time of 1.5-2 hours, 36% of respondents reported visits completed between 1-1.5 hours, and 11% of respondents reported visit times under one hour.

Patients reported a more positive overall clinic experience when seen by primary care physicians, when they had been a patient for less than six months, when they had an appointment that lasted more than one hour, and when they were male, compared to their counterparts. Patient satisfaction was not affected by whether the patient received any free medications.

The free text responses regarding clinic strength were all positive. Patients felt the clinic had, “very professional and caring folks.” They also felt, “everyone is friendly, loving, caring, treat patients with great respect.” Another response indicated, “one big benefit is that students pick up every hint and nuance of any issue I mention. They really listen well. I barely mentioned past experience with skin cancers and they later brought me sunscreen.” The free text responses to ‘what could we do to serve you better’ stated “… appointments a bit shorter; But that is the tiniest issue - not really a worry at all …”, and “time … waiting between … med students and Dr. …[shorten] to one hour would be better.”

## Discussion

This study indicated that most of the KNIGHTS clinic patients would recommend this clinic to a friend or relative and were either “satisfied” or “very satisfied” with all aspects of the clinic. The patients reported high satisfaction with students’ and providers’ knowledge. Most importantly, patients receiving care at the KNIGHTS Clinic felt invested in the training of future healthcare providers. This finding may strengthen and increase the future provider's and medical school’s commitment to providing healthcare through SRFCs.

As the highest rated parameters were “availability of free/affordable medicine”, “primary/specialty care”, and “team approach”, other SRFCs looking to improve their patient satisfaction can utilize or add or improve these aspects. “Courtesy and respect of staff” was another highly rated factor, showing that patients value the care and attention to detail received from students and faculty members. The current medical school curriculum at UCF College of Medicine strongly emphasizes expression of empathy towards patients and developing a personable relationship with patients, which translated to student physician care at SRFCs. Physician empathy and the development of the patient-physician relationship is strongly stressed in many medical school curriculums and can be utilized in the SRFC setting to further improve the overall clinic experience. Patient wait times and length of stay received positive ratings but were the lowest compared to other assessment items. This is an area that needs to be improved across all aspects of medicine, including SRFCs. Despite the wait time, patients still rated their overall satisfaction with the clinic very highly. This could possibly be due to the variety of care provided during the visit. To improve the wait times and length of stays at KNIGHTS clinic, several quality improvement interventions will be implemented to strengthen these weaknesses.

One of the main limitations of this study is the relatively small sample size. Additionally, there may be a self selection bias in the patient population that responded to our questionnaire. Patients who responded were probably more inclined if they were extremely satisfied or dissatisfied with their care at the clinic. There was no selection bias for our patient population, as all patients were offered an opportunity to complete the survey.

SRFCs across the nation are thriving and increase in number every year. We hope by sharing our data that other SRFCs can learn from our results and further improve their own patient care. Additionally, we hope that the developed survey can be utilized at SRFCs nationally to help standarize the evaluation of care received at SRFCs.

## Conclusions

This study used a content validated survey to show that the KNIGHTS Clinic received positive feedback with an overall highly rated patient satisfaction and perception of the care received, with the most appreciated services being the availability of specialty care and free services and the opportunity to influence the education of future physicians. These results will act as a baseline for quality improvement and interventions at the KNIGHTS Clinic. We hope this survey may be utilized at SRFCs across the nation as a standardized survey to evaluate the current environment of SRFCs. The KNIGHTS Clinic will continue to monitor the care received at our SRFC, and we hope that by showing the vital healthcare that SRFCs provide to local communities, we may further expand our patient population.

## Appendices

**Table 2:** Keeping Neighbors in Good Health Through Service (KNIGHTS) Clinic patient satisfaction survey questions Each item response allowed for answers between 1-5, 1 being very unsatisfied and 5 being very satisfied.

How satisfied are you with the following aspects of this clinic visit? Availability of free and/or affordable medications Availability of both primary care and specialists (such as cardiology, nephrology, etc.) Education on disease treatment and prevention Education about your medications Wait time during this appointment Length of time spent in the clinic from arrival to departure Overall medical care Students’ knowledge Doctors’ knowledge Treatment by teaching faculty Availability of after-hours appointments Courtesy and respect of our staff Team approach to your medical care including pharmacy/medical students partnering with doctors Overall clinic experience
How satisfied are you with the improvement in your health since you first came to the evening KNIGHTS Clinic?
One of the benefits of the evening KNIGHTS Clinic is the opportunity to influence the education of future physicians. How much do you value this aspect?
What do you think the evening KNIGHTS Clinic does well?
How can the evening KNIGHTS Clinic serve you better?
Age
Gender
Today, did you see a specialist (i.e. cardiology, etc.) or a primary care physician?
How long ago did you first come to an evening KNIGHTS Clinic?
How long was the total time of your visit today, including waiting time?
Did you receive free medication (samples or medicines through the drug assistance programs)?
Were you seen by a member of the patient education team (a student who educates you about health or medicines after the history and physical exam portion of your clinic visit)?
Did you use the lab today for services including blood draws, vaccinations, or urine samples?
Have you been seen at the evening KNIGHTS Clinic within the past month?
